# Noncoding RNAs and the phytochemical economy: Molecular regulators of secondary metabolism in medicinal plants

**DOI:** 10.1016/j.bbrep.2026.102486

**Published:** 2026-02-09

**Authors:** Okechukwu Paul-Chima Ugwu, Melvin Nnaaemeka Ugwu, Hope Onohuean, Hilal Ahmad Rather, Ibe Michael Usman

**Affiliations:** a Department of Research, Publication and Extension, Kampala International University, Kampala, Uganda; b Department of Medical Biochemistry, Faculty of Basic Medical Sciences, State University of Medical and Applied Science, Igbo Eno, Enugu, Nigeria; c Biopharmaceutics Unit, Department of Pharmacology and Toxicology, School of Pharmacy, Kampala International University Uganda, Ishaka-Bushenyi, Western Campus, Uganda; d Department of Biochemistry Kampala International University Uganda, Western Campus, Uganda; e Department of Human Anatomy Faculty of Biomedical Science Kampala International University, Uganda

**Keywords:** Noncoding RNAs, Secondary metabolism, Medicinal plants, Metabolic engineering, RNA-Mediated epigenetic control, RNA-Directed DNA methylation (RdDM)

## Abstract

**Background:**

Medicinal plants produce specialised (secondary) metabolites including alkaloids, terpenoids, flavonoids and phenolics that underpin pharmaceuticals, nutraceuticals and traditional therapeutics. Although enzyme-encoding genes and transcription factors are established regulators of these pathways, accumulating evidence indicates that noncoding RNAs (ncRNAs) provide additional, and in some contexts decisive, regulatory control.

**Objective:**

To synthesise and critically appraise evidence on how plant ncRNAs microRNAs (miRNAs), small interfering RNAs (siRNAs), long noncoding RNAs (lncRNAs) and circular RNAs (circRNAs) regulate secondary metabolism in medicinal plants, and to map translational opportunities and unresolved gaps.

**Methods:**

We conducted a narrative review with a systematic synthesis across PubMed/MEDLINE, Scopus, Web of Science Core Collection, CAB Abstracts and AGRICOLA, supplemented by Google Scholar screening using SANRA.

**Results:**

Across medicinal and non-model plant systems, miRNAs repeatedly target transcription factors that control biosynthetic pathways (e.g., MYB/bHLH/WRKY families) and, in some cases, key enzymes (e.g., PAL/CHS/DFR modules), thereby supporting stress-responsive and developmentally timed reprogramming of metabolite profiles. siRNAs contribute through RNA-directed DNA methylation (RdDM) and regulation of transposons and gene clusters. lncRNAs modulate chromatin accessibility and function as scaffolds or decoys, including as competing endogenous RNA (ceRNA) ‘sponges’, whereas circRNAs are emerging as relatively stable regulatory hubs that may influence miRNA availability and stress-associated transcriptional states. Evidence quality varies across ncRNA classes and species, and mechanistic validation in medicinal plants remains inconsistent.

**Conclusions:**

ncRNAs constitute a multilayer regulatory system shaping the ‘phytochemical economy’ of medicinal plants. Progress towards translation will require standardised ncRNA annotation resources, rigorous causal validation, and integrated multi-omics study designs to support precision metabolic engineering and sustainable phytochemical production.

## Highlights

• NcRNAs (miRNAs, siRNAs, lncRNAs, circRNAs) regulate specialised metabolism.
• Evidence supports ncRNA control of MYB/bHLH/WRKY TFs and key enzymes in pathways.
• LncRNA/circRNA ceRNA networks and chromatin remodelling shape tissue metabolites.
• Gaps: limited validation in medicinal species, ncRNA databases, and causal omics.

## Graphical abstract

**Figure:**
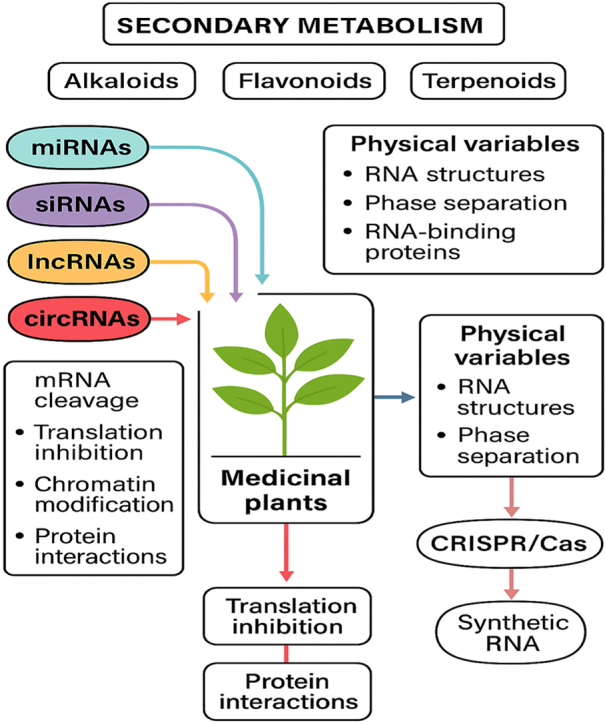

## 1 Introduction

### 1.1 Background

Medicinal plants remain a major source of bioactive compounds for modern and traditional therapeutics because they produce structurally diverse specialised metabolites (e.g., alkaloids, flavonoids, terpenoids and phenolics) [1,2]. This pharmacological relevance has sustained interest in how plants allocate metabolic flux to pathway-specific products under developmental and environmental constraints [3,4]. Two recent syntheses further emphasise the clinical and translational relevance of medicinal-plant metabolites for therapeutic development and related bioorganic applications [5,6].

Secondary metabolites, unlike primary metabolites required for basal growth, mediate defence, signalling, ecological interactions and stress adaptation, thereby contributing to medicinal value and variability in phytochemical accumulation [2,3,6]. Consequently, classical models of pathway regulation have emphasised enzyme-encoding genes and transcription-factor hierarchies (e.g., MYB/bHLH/WRKY), enabling metabolic engineering through overexpression or knockout strategies [[5], [6], [7], [8]]. However, these gene-centric frameworks do not fully explain tissue specificity, rapid stress-induced metabolic reprogramming, transgenerational memory, or the frequent dissociation between transcript abundance and metabolite output [[9], [10], [11], [12], [13]].

### 1.2 Rationale

Noncoding RNAs (ncRNAs) regulate gene expression at post-transcriptional and epigenetic levels. In plants, microRNAs (miRNAs) and small interfering RNAs (siRNAs) mediate sequence-specific mRNA cleavage and translational repression and contribute to epigenetic silencing via RNA-directed DNA methylation (RdDM). Long noncoding RNAs (lncRNAs) and circular RNAs (circRNAs) provide additional regulatory control through chromatin modulation, RNA-protein scaffolding and competing endogenous RNA (ceRNA) networks [10,14]. The ncRNA classes considered here (miRNAs, siRNAs, lncRNAs and circRNAs) are consistent with broader ncRNA biology frameworks and recent mechanistic syntheses [[15], [16], [17], [18], [19], [20], [21], [22], [23]]. For definitional clarity, we support each ncRNA class at first mention: (i) miRNAs and their regulatory scope [24]; (ii) siRNA biology and genome defence/RdDM [[15], [16], [17]]; (iii) lncRNA definitions and functional recommendations [18,19]; and (iv) circRNA regulatory roles and emerging systems relevance [[20], [21], [22], [23]].

### 1.3 Aim and scope

**Aim:** This review synthesises and critically appraises evidence on how plant ncRNAs regulate secondary metabolism in medicinal plants, with emphasis on (1) mechanistic modes (post-transcriptional, epigenetic and network-based control), (2) pathway-level targets (transcription factors, enzymes, transport, and cluster regulation), and (3) applied opportunities in metabolic engineering, breeding and synthetic biology.

**Scope decisions:** We prioritise 2015–2025 because this period captures rapid expansion in high-throughput small-RNA sequencing, improved ncRNA annotation and multi-omics integration in plant specialised metabolism; however, we include older seminal studies where required to establish foundational mechanistic context.

Schematic overview of how ncRNAs regulate secondary metabolite production in medicinal plants. Major ncRNA classes microRNAs (miRNAs), small interfering RNAs (siRNAs) and long noncoding RNAs (lncRNAs) act as key regulators of pathways that underpin metabolite diversity (e.g., flavonoids and alkaloids). ncRNAs modulate gene expression through several mechanisms, including mRNA cleavage, translational inhibition, chromatin modification, DNA methylation and stress-responsive regulation. Together, these regulatory activities provide opportunities for metabolic engineering to optimise secondary metabolite biosynthesis. **Abbreviations:** ncRNA, noncoding RNA; miRNA, microRNA; siRNA, small interfering RNA; lncRNA, long noncoding RNA; mRNA, messenger RNA.

## 2 Methodology

### 2.1 Review design and methodological framework

We conducted a systematised narrative review using a transparent and reproducible literature search combined with structured qualitative synthesis to integrate heterogeneous mechanistic evidence on non-coding RNA (ncRNA) regulation of specialised (secondary) metabolism in medicinal plants. We guided the methodological quality and reporting of the narrative synthesis using the SANRA (Scale for the Assessment of Narrative Review Articles) framework, which prioritises explicit objectives, systematic literature identification, transparent data handling and balanced interpretation.

### 2.2 Information sources and search scope

We searched PubMed/MEDLINE, Scopus, Web of Science Core Collection, CAB Abstracts (CABI) and AGRICOLA to capture literature spanning molecular plant biology and applied phytochemistry. To mitigate publication bias and improve coverage of emerging mechanistic studies, we additionally screened Google Scholar (first 200 results per query string) and manually examined reference lists of high-relevance reviews and seminal experimental articles.

### 2.3 Temporal scope and rationale

We focused on studies published between 2015 and 2025, reflecting the rapid growth of medicinal-plant ncRNA research, circRNA discovery pipelines and integrative multi-omics approaches during this period. We included earlier studies selectively when they were foundational for ncRNA biogenesis, RNA-directed DNA methylation (RdDM) pathways or early conceptual models linking gene regulation to specialised metabolism, and when they were necessary for conceptual continuity.

### 2.4 Search strategy

We applied a structured Boolean strategy combining ncRNA-related terms, specialised-metabolism terminology and a medicinal-plant or biotechnology context. Core terms encompassed ‘non-coding RNA’, ‘microRNA’, ‘small interfering RNA’, ‘long non-coding RNA’, ‘circular RNA’, ‘secondary/specialised metabolism’, major phytochemical classes (including alkaloids, flavonoids, terpenoids and phenylpropanoids) and descriptors related to plant biotechnology or metabolic engineering. We adapted search strings to individual database requirements, and full search strategies are reported in Supplementary Table S1.

### 2.5 Study selection and transparency

We exported all retrieved records to a reference management system and removed duplicates before screening. We assessed studies in two stages, comprising title and abstract screening followed by full-text evaluation against predefined eligibility criteria. Supplementary Table S2 reports the numbers of records screened, assessed and included, together with reasons for exclusion at the full-text stage, ensuring transparent evidence selection without presenting the review as a formal systematic meta-analysis.

### 2.6 Eligibility criteria and evidence grading

To support structured synthesis and minimise subjective selection, we applied an operational evidence-grading rubric based on relevance and methodological rigour. Relevance reflected the extent to which studies established mechanistic links between ncRNA(s) and specialised-metabolism outcomes, ranging from direct experimental effects on metabolite accumulation or pathway flux to indirect regulatory associations. Rigour captured the strength of molecular validation, distinguishing experimentally confirmed ncRNA-target interactions supported by metabolite-level evidence from correlational or predictive multi-omics analyses. We retained studies meeting minimum relevance and rigour thresholds and assigned greater interpretive weight to those providing direct mechanistic validation.

### 2.7 Quality appraisal using SANRA principles

Consistent with SANRA guidance for narrative reviews, we appraised studies qualitatively rather than excluding them solely on study design. We focused on clarity of objectives, appropriateness of methods, transparency of molecular validation, adequacy of metabolite measurement and balance of interpretation.

### 2.8 Data extraction and synthesis

From each included study, we extracted data on plant species and tissue, metabolite class or pathway, ncRNA type, predicted and/or validated targets, experimental validation approaches, genetic or molecular perturbation strategies, metabolite assay methods, developmental or stress context and reported limitations. We synthesised evidence thematically using a hybrid deductive–inductive approach, organising studies into mechanistic domains such as miRNA-mediated transcription factor regulation, siRNA/RdDM pathways, lncRNA-associated chromatin modulation, competing endogenous RNA networks and translational or engineering-relevant mechanisms, while allowing novel or species-specific patterns to emerge.

### 2.9 Bias mitigation and interpretive balance

To reduce confirmation bias, we incorporated search terms designed to capture neutral or negative findings and explicitly reported contradictory or non-reproducible results where present. We constrained interpretations by study quality and clearly distinguished experimentally validated ncRNA-target relationships from associations inferred solely from expression profiling or computational prediction. We avoided overgeneralisation across species or metabolite classes by contextualising conclusions within the experimental systems examined.

### 2.10 Methodological limitations

The evidence base spans diverse plant taxa, tissues, ncRNA classes, experimental platforms and metabolite endpoints, which precludes quantitative synthesis or formal meta-analysis. Accordingly, conclusions are evidence-weighted and mechanistically focused but remain interpretive where causal validation is incomplete. We explicitly acknowledge these limitations to align with SANRA criteria for balanced and critical narrative synthesis.

### 2.11 Evidence key used in the tables

**Tier A (causal):** ncRNA perturbation (OE/KO/editing/mimic) + validated target + metabolite/flux phenotype.

**Tier B (mechanistic-partial):** validated targeting and/or genetic evidence with metabolic association, but incomplete causal chain.

**Tier C (associative):** expression/profiling, prediction, co-expression, or network inference without causal perturbation.

### 2.12 Overview of noncoding RNAs in plants

Noncoding RNAs (ncRNAs) are RNA molecules that do not encode proteins but exert important regulatory effects on gene activity at transcriptional, post-transcriptional and epigenetic levels [14]. In plants, ncRNAs are increasingly recognised as regulators of growth, stress responses and metabolic processes, including secondary metabolism [15]. Plant ncRNAs are commonly grouped into four classes on the basis of size, structure and function: microRNAs (miRNAs), small interfering RNAs (siRNAs), long noncoding RNAs (lncRNAs) and circular RNAs (circRNAs) (Table 2) [16]. These classes differ in biogenesis and mode of action, together contributing to the layered control of gene expression in plant cells [17].

**Table 1:** Specialised metabolite classes in medicinal plants and regulatory layers (TFs and ncRNAs).

Metabolite class (examples)	Core plant function(s)	Representative medicinal relevance (examples)	Dominant regulatory nodes (pathway level)	Representative ncRNA modules linked to pathway control	Evidence
**Flavonoids/anthocyanins** (quercetin, kaempferol, anthocyanins)	UV protection, pigmentation, antioxidant defense	Antioxidant, anti-inflammatory, vasoprotective	**MBW complex** (MYB-bHLH-WD40); CHS/CHI/F3H/DFR/ANS branches	**miR156→SPL** (anthocyanin/flavonoid programs); **miR858→MYBs** (flavonoid/phenylpropanoid regulators); **miR828/TAS4→MYB network**	A-B
**Phenylpropanoids** (lignin precursors, phenolic acids, tannins)	Structural support, pathogen resistance, oxidative buffering	Antioxidant, antimicrobial, anti-inflammatory	PAL/C4H/4CL; R2R3-MYBs; chromatin state of pathway loci	**miR858→MYB TFs** shifts lignin–flavonoid allocation; lncRNA-guided chromatin modulation reported in medicinal species (multi-omics)	A-C
**Terpenoids** (artemisinin, taxanes, mono/diterpenes)	Defense, volatile signaling, herbivore deterrence	Antimalarial (artemisinin), anticancer (taxanes)	MEP/MVA flux; terpene synthases; jasmonate TF networks	Multiple miRNAs implicated in *A. annua* artemisinin networks (mostly profiling/prediction in medicinal plants)	C
**Alkaloids** (terpenoid indole alkaloids; morphinan-type etc.)	Potent defense/anti-herbivory	Analgesic/anticancer/antimicrobial (class-dependent)	Pathway TF hubs; BGC/clustered loci (where present)	ncRNA control proposed via TF targeting + RdDM (limited medicinal-plant causality)	C
**Biosynthetic gene clusters (BGCs)** (subset of specialised pathways)	Coordinated pathway expression	Enables pathway-level engineering targets	Chromatin state, transposon control, RdDM	**siRNA/RdDM** and TE control can influence cluster accessibility/expression (conceptually strong; species-specific validation variable)	B–C

Table 1: *Compiled and adapted from* Refs. [25,26]**.** Examples are illustrative, not exhaustive. Use Evidence tiers to prevent overgeneralization across taxa.

**Table 2:** Plant ncRNA classes: biogenesis, mechanisms, and relevance to specialised metabolism.

ncRNA class	Typical size/structure	Core biogenesis (plants)	Primary mechanisms	Specialised-metabolism relevance	Evidence base in medicinal plants
**miRNA**	∼21 nt; single-stranded	Pol II pri-miRNA → DCL1 processing → AGO1-RISC loading	Target cleavage/translational repression	Frequently targets **TF hubs (MYB/bHLH/SPL/WRKY);** occasional direct enzyme targeting	Stronger (A-B in several systems)
**siRNA**	∼21–24 nt; duplex	dsRNA → DCLs; RDRs; AGO (AGO4/6/9 in RdDM context)	PTGS**; RdDM;** heterochromatin formation	Can regulate **TEs,** genomic stability, and potentially cluster/proximal regulation of specialised loci	Moderate, often indirect (B–C)
**lncRNA**	>200 nt; linear; often spliced	Mostly Pol II; diverse processing; low sequence conservation	Scaffold/decoy; chromatin recruitment; ceRNA; transcriptional modulation	Reported as regulators in medicinal plants via network integration and TF/enzymatic modules (causal cases still limited)	Emerging (B–C)
**circRNA**	covalently closed loop	back-splicing; resistant to exonucleases	miRNA/RBP sponge; transcriptional effects; possible R-loops	Reported in medicinal species**; genome-wide circRNA catalogs** linked to secondary metabolite pathways; limited causal functional tests	Emerging (B–C; rare A)

**Fig. 1:**
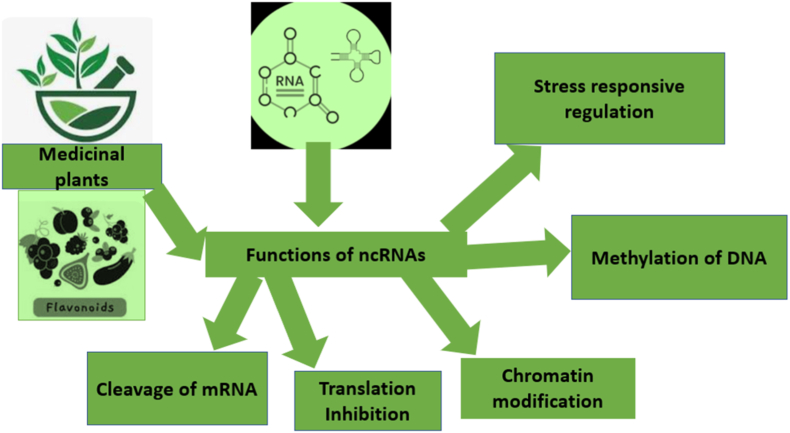
Regulatory roles of noncoding RNAs (ncRNAs) in secondary metabolism of medicinal plants.

Table 2: *Compiled and adapted from* Refs. [10,[14], [15], [16], [17],^27^]. In plants, circRNA “miRNA sponge” is often computational unless genetically tested; treat claims accordingly (see Fig. 1).

microRNAs (miRNAs) are single-stranded RNAs of approximately 21 nucleotides that originate from hairpin precursors transcribed by RNA polymerase II [18]. After nuclear processing by Dicer-like (DCL) enzymes, mature miRNAs are incorporated into the RNA-induced silencing complex (RISC), which guides sequence-specific binding to target mRNAs and typically induces mRNA cleavage or translational repression [19]. Through these mechanisms, miRNAs regulate transcription factors, enzymes and other regulatory proteins involved in plant development, hormone signalling and stress adaptation [18].

Small interfering RNAs (siRNAs) are of similar length to miRNAs but are typically derived from longer double-stranded RNA precursors, including exogenous RNA and endogenous repetitive sequences, through DCL-dependent processing [19]. In association with RISC, siRNAs promote sequence-specific mRNA degradation and can also mediate transcriptional silencing via RNA-directed DNA methylation (RdDM) [20]. These activities contribute to defence against transposons and viral RNAs and support genome stability [19].

Long noncoding RNAs (lncRNAs) are a heterogeneous group of transcripts longer than 200 nucleotides with limited protein-coding potential [20]. lncRNAs regulate gene expression through multiple mechanisms, including chromatin remodelling, RNA–DNA triplex formation, RNA-protein interactions and molecular scaffolding [21]. They can recruit chromatin-modifying complexes, influence histone modifications and modulate three-dimensional genome organisation [21]. In addition, some lncRNAs act as precursors of small RNAs or function as molecular decoys for RNA-binding proteins or miRNAs, thereby reshaping gene regulatory networks [22].

Circular RNAs (circRNAs) are covalently closed RNAs generated by back-splicing of precursor mRNAs [23]. Their circular structure confers resistance to exonuclease-mediated degradation, contributing to increased stability [27]. circRNAs can sequester miRNAs and thereby participate in competing endogenous RNA networks [27]. Although functional characterisation of plant circRNAs remains limited, available evidence indicates roles in development, stress responses and secondary metabolism, potentially through modulation of miRNA availability [23].

Biogenesis of plant ncRNAs is tightly regulated and involves diverse enzymes and cofactors. For miRNAs and siRNAs, this includes coordinated actions of DCL proteins, RNA-dependent RNA polymerases (RDRs) and Argonaute proteins [27]. RNA polymerase II transcribes lncRNAs and circRNAs, which undergo capping, splicing and polyadenylation (except circRNAs), and may also arise through alternative splicing pathways [28,29]. ncRNA stability, subcellular localisation and transport further shape regulatory activity [30]. miRNAs and siRNAs are often stabilised by 3′-end methylation, whereas lncRNAs and circRNAs exhibit structural features that can increase resistance to degradation [31]. RNA-binding proteins and localisation signals mediate trafficking between cellular compartments and, in some cases, intercellular movement via plasmodesmata or extracellular vesicles [32]. Such transport supports coordination of gene expression across tissues, particularly during developmental transitions and stress responses [33]. Collectively, plant ncRNAs form an integrated regulatory system that coordinates gene expression through sequence-specific interactions, epigenetic mechanisms and intercellular communication [33].

### 2.13 miRNAs in the regulation of secondary metabolism

**Fig. 2:**
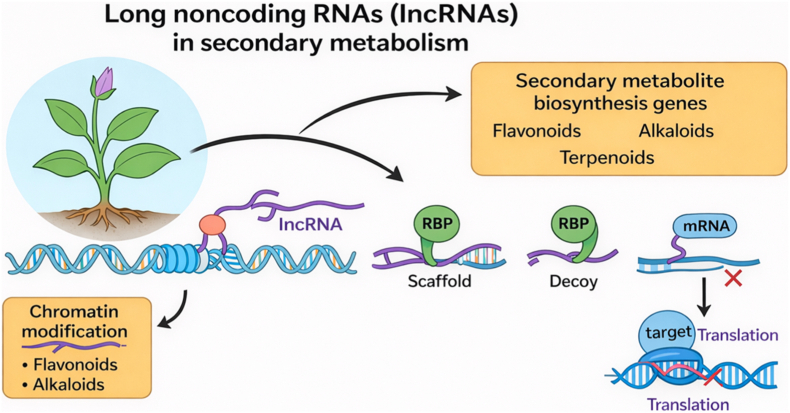
Long noncoding RNAs (lncRNAs) regulate plant secondary metabolism.

MicroRNAs (miRNAs) mediate post-transcriptional gene regulation in plants, and their roles in secondary metabolism provide an important mechanism by which plants adjust bioactive compound production in response to developmental cues and environmental stimuli (Fig. 2; Table 3) [33]. A major route by which miRNAs influence secondary metabolism is through regulation of transcription factors that coordinate entire biosynthetic pathways [33]. miRNAs target transcription factors such as MYB (myeloblastosis), bHLH (basic helix-loop-helix) and WRKY, which act as master regulators of genes involved in the biosynthesis of flavonoids, alkaloids, terpenoids and phenylpropanoids [34]. By fine-tuning transcription factor abundance, miRNAs indirectly modulate the activation or repression of multiple downstream biosynthetic genes.

**Table 3:** miRNA modules repeatedly implicated in specialised metabolism.

miRNA module	Primary validated target(s)	Pathway/process impacted	Species (examples)	Key experimental support	Evidence
**miR156 → SPLs**	SPL TFs (e.g., SPL9/others)	Anthocyanin/flavonoid programs; developmental timing–metabolism coupling	*Arabidopsis*; conserved across plants	Target validation; genetic perturbation; metabolite/pigment phenotypes	**A**
**miR858 → R2R3-MYBs**	MYB TFs controlling flavonoid/phenylpropanoid genes	Flavonoid branch and lignin/flavonoid allocation	*Arabidopsis* (validated); broader plant reports	Overexpression/mimicry; MYB downregulation; flavonoid changes	**A**
**miR828/TAS4 siRNAs → MYB network**	MYB regulators (e.g., VvMYB114; anthocyanin MYBs)	Anthocyanin/flavonoid feedback control	Grapevine; *Arabidopsis* network	sRNA pathway + MYB regulation; anthocyanin outcomes	**A-B**
**Artemisinin-linked miRNA sets (multiple)**	Enzymes/TFs in artemisinin network (often predicted)	Terpenoid (artemisinin) pathway regulation hypothesis	*Artemisia annua*	Mostly sRNA profiling + target prediction; limited causal evidence	**C**

Table 3: *Compiled and adapted from* Refs. [[33], [34], [35], [36], [37], [38], [39], [40], [41], [42], [43], [44], [45]].

In addition to transcription factor regulation, some miRNAs directly target transcripts encoding key metabolic enzymes [34]. miRNAs targeting chalcone synthase (CHS), phenylalanine ammonia-lyase (PAL) and flavanone 3-hydroxylase (F3H) enzymes central to the flavonoid and phenylpropanoid pathways have been reported across diverse plant species [35]. Such direct repression can alter pathway flux and provides a rapid mechanism for adjusting secondary metabolite levels in response to internal and external signals [34,35]. These interactions contribute to the plasticity of metabolite production, which supports adaptation to changing environments.

miRNA profiles also respond dynamically to abiotic and biotic stress, with consequences for secondary metabolite composition [34]. Under drought, salinity, temperature variation and pathogen challenge, shifts in miRNA expression can redirect metabolism towards defence-associated secondary metabolites [36]. These changes may enhance stress tolerance while altering the abundance and composition of medicinally relevant compounds, underscoring the coupling between stress responses and secondary metabolism.

Several well-characterised modules illustrate the specificity and mechanistic breadth of miRNA control over secondary metabolism [37,38]. The miR156-SPL (SQUAMOSA PROMOTER BINDING PROTEIN-LIKE) module is among the most extensively studied: miR156 represses SPL transcription factors that influence flavonoid biosynthesis genes, including dihydroflavonol 4-reductase (DFR) and leucoanthocyanidin dioxygenase (LDOX) [39,40]. This regulation is developmentally timed and environmentally responsive, indicating how miRNAs integrate multiple cues to shape metabolite biosynthesis. Another example is miR858, which targets several MYB transcription factors that regulate the phenylpropanoid pathway responsible for lignin, flavonoids and other phenolic compounds [40]. Manipulation of miR858 abundance has been shown to modify lignin content and flavonoid profiles, supporting its potential utility in metabolic engineering [41].

Evidence from medicinal plants further highlights context-dependent roles for miRNAs in secondary metabolism [42]. In *Artemisia annua*, miRNAs have been associated with terpenoid biosynthesis and trichome development, both relevant to artemisinin production [43]. In *Salvia miltiorrhiza*, multiple miRNAs modulate genes involved in rosmarinic acid and salvianolic acid biosynthesis, often in coordination with hormone signalling pathways [44]. In *Catharanthus roseus*, which produces the anticancer alkaloids vincristine and vinblastine, miRNAs regulate pathway genes such as strictosidine synthase (STR) and geraniol 10-hydroxylase (G10H), thereby influencing the balance between growth and alkaloid accumulation [45]. Together, these studies support a central role for miRNAs in secondary metabolism through both indirect (transcription factor-mediated) and direct (enzyme-targeting) mechanisms [[42], [43], [44], [45]].

Overall, miRNAs function as context-dependent regulators that shape metabolite accumulation by modulating biosynthetic genes and their upstream controllers. Defining these regulatory relationships will advance understanding of plant metabolic control and inform strategies to optimise the production of high-value secondary metabolites in medicinal plants.

Schematic overview of representative lncRNA-mediated mechanisms that regulate secondary metabolite biosynthesis. lncRNAs can act at the chromatin level by recruiting or modulating chromatin-associated factors, thereby altering transcription of secondary metabolite biosynthetic genes (e.g., loci associated with flavonoid and alkaloid biosynthesis). In the nucleus and/or cytoplasm, lncRNAs may function as **scaffolds** to assemble RNA-binding proteins (RBPs) into regulatory complexes, or as **decoys** that sequester RBPs away from their canonical RNA targets. In the cytoplasm, lncRNAs may also inhibit gene expression post-transcriptionally by interfering with **target mRNA** translation. **Abbreviations:** lncRNA, long noncoding RNA; RBP, RNA-binding protein; mRNA, messenger RNA.

### 2.14 lncRNAs as epigenetic and post-transcriptional regulators

Long noncoding RNAs (lncRNAs) are transcripts longer than 200 nucleotides with limited protein-coding potential and are increasingly recognised as important regulators of gene expression in plants [46]. They influence secondary metabolism through epigenetic and post-transcriptional mechanisms, thereby modulating biosynthetic pathways that underpin the production of medicinally relevant phytochemicals [47]. A defining feature of lncRNAs is their capacity to reshape gene expression programmes in response to developmental and environmental cues (Table 4).

**Table 4:** lncRNA mechanisms relevant to specialised metabolism.

Mechanistic role	Molecular mechanism	Expected impact on specialised metabolism	Example evidence in medicinal/non-model plants	Evidence
**Chromatin recruitment/epigenetic modulation**	lncRNA recruits chromatin modifiers (repressive or activating marks) to loci	Coordinated on/off switching of pathway genes; potential BGC regulation	Multi-omics studies in *Salvia miltiorrhiza* implicate lncRNA-mRNA-TF networks in secondary-metabolite pathways	B–C
**Scaffold for RNP complexes**	lncRNA binds RBPs/TFs to assemble regulatory complexes	Stabilizes or organizes expression programs under stress/development	Plant systems broadly; medicinal-plant mechanistic depth still limited	C
**Decoy (“molecular sink”)**	lncRNA sequesters TFs/RBPs or miRNAs	Rapid rebalancing of pathway regulators during stress	Reported widely as a mechanism; medicinal plant causality rare	C
**ceRNA/miRNA sponge**	lncRNA contains miRNA response elements; competes with mRNAs	De-represses pathway TFs/enzymes by titrating miRNAs	Commonly inferred from networks; validate via perturbation + rescue	C

Table 4: *Compiled and adapted from* Refs. [27,45,[48], [49], [50], [51], [52], [53], [54], [55], [56], [57]].

One well-established mode of action involves changes in chromatin accessibility at loci encoding secondary metabolite biosynthetic genes [58]. Some lncRNAs recruit chromatin-modifying complexes, including histone methyltransferases and acetyltransferases, to specific genomic regions, altering chromatin states to repress or activate transcription [48]. This epigenetic regulation contributes to the temporal and spatial control of metabolic pathway activity [48]. For example, lncRNA-mediated chromatin remodelling can increase or restrict access of the transcriptional machinery to genes encoding enzymes in flavonoid, alkaloid or terpenoid biosynthesis, with consequent effects on metabolite abundance [49].

Beyond chromatin-level regulation, lncRNAs often function as molecular scaffolds or decoys for RNA-binding proteins (RBPs). As scaffolds, they can assemble RBPs and/or transcription factors into ribonucleoprotein complexes that influence mRNA stability, translation and nuclear export of biosynthetic transcripts [50]. As decoys, lncRNAs can sequester RBPs away from their target mRNAs, thereby altering gene expression by limiting the availability of regulatory proteins [51]. This mechanism may be particularly relevant under stress, when rapid reconfiguration of metabolic outputs supports production of defence-associated compounds.

lncRNAs also participate in competing endogenous RNA (ceRNA) networks by binding microRNAs (miRNAs) and reducing their availability to repress cognate mRNA targets [52]. In these lncRNA–miRNA–mRNA axes, sequence complementarity enables lncRNAs to sequester miRNAs, indirectly increasing expression of miRNA-targeted transcripts [52]. This additional layer of post-transcriptional control may enhance the flexibility of metabolite biosynthesis. For example, sequestration of a miRNA that targets a phenylpropanoid-related transcription factor or enzyme could relieve repression and increase pathway output, thereby elevating the accumulation of lignins, flavonoids or stilbenes.

Emerging evidence also implicates lncRNAs in stress-induced phytochemical reprogramming, highlighting their role in dynamic metabolic responses [53]. Several lncRNAs show marked changes in expression under abiotic stresses (e.g., drought, salinity and temperature extremes) and biotic challenges (e.g., pathogen infection), and can drive downstream transcriptional and epigenetic changes that selectively activate biosynthetic genes involved in stress-mitigating secondary metabolites. For instance, in *Arabidopsis thaliana*, specific lncRNAs are induced under oxidative stress and influence genes in the glucosinolate pathway, whereas in *Salvia miltiorrhiza* stress-responsive lncRNAs have been linked to regulation of tanshinone production [54].

An additional area of interest is the potential contribution of lncRNAs to the regulation of biosynthetic gene clusters (BGCs) [55]. BGCs comprise co-localised genes encoding enzymes for entire biosynthetic pathways, and coordinated expression is required for efficient metabolite production [56]. Although plant BGC regulation is less well characterised than in microbes, accumulating evidence suggests that lncRNAs may influence BGC activity through chromatin-based mechanisms or by promoting long-range chromatin interactions that support coordinated transcription [57]. For example, lncRNAs may act as architectural components that bring distal regulatory elements into proximity with BGC promoters, facilitating co-regulation of pathway genes.

In summary, lncRNAs provide a flexible layer of regulatory control over secondary metabolism by integrating chromatin remodelling, RBP interactions and ceRNA networks. Their emerging roles in stress responsiveness and potential regulation of BGCs highlight their relevance to plant physiology and to metabolic engineering aimed at enhancing production of pharmacologically important compounds. Further mechanistic studies will be required to clarify how lncRNA-centred regulatory networks organise specialised metabolism in medicinal plants.

### 2.15 Circular RNAs and their emerging role

Circular RNAs (circRNAs) are endogenous noncoding RNAs characterised by a covalently closed loop generated through a non-canonical splicing event termed back-splicing [59]. Unlike linear RNAs, circRNAs lack 5′ caps and 3′ poly(A) tails, which confers resistance to exonuclease-mediated degradation and contributes to high stability [60]. Their stability and tissue- and developmental stage-specific expression patterns suggest potential regulatory roles in plant gene expression, including pathways governing secondary metabolism in medicinal plants (Table 5).

**Table 5:** circRNAs in medicinal plants: features, functional hypotheses, and current evidence.

Feature	What it means mechanistically	Relevance to specialised metabolism	Representative plant evidence	Evidence
**High stability (closed loop)**	resistant to exonucleases; often tissue/stress specific	Attractive as durable regulatory hubs/biomarkers	Broad plant circRNA catalogs; stress-linked circRNAs common	B–C
**Genome-wide discovery in medicinal species**	RNA-seq + circRNA callers; functional inference by networks	Allows pathway-linked circRNA prioritization	*Salvia miltiorrhiza* circRNAs potentially involved in secondary metabolite biosynthesis	C
**miRNA sponge (validated in plants, rare)**	circRNA sequesters miRNA; can be genetically tested	Can indirectly elevate enzymes/TFs in pathways	First genetic evidence for plant circRNA acting as miRNA sponge via CRISPR deletion of circRNA loci	**A (rare)**
**RBP interaction/transcriptional effects**	scaffolding or regulation at transcriptional level	May coordinate stress-induced pathway reprogramming	Mostly review-level inference in plants; validate case-by-case	C

Table 5: *Compiled and adapted from* Refs. [55,56,[59], [60], [61], [62], [63], [64], [65], [66], [67], [68], [69]].

circRNA biogenesis involves fusion of a downstream 5′ splice donor to an upstream 3′ splice acceptor, typically facilitated by complementary sequences and/or RNA-binding proteins that juxtapose splice sites [59]. Back-splicing can generate exonic circRNAs, intronic circRNAs, or exon–intron circRNAs. Although plant circRNAs are less comprehensively identified and characterised than their animal counterparts, advances in high-throughput RNA sequencing and computational detection have revealed an expanding repertoire in plants, including medicinal species such as *Salvia miltiorrhiza*, *Catharanthus roseus* and *Panax ginseng* [61,62].

A frequently investigated function of circRNAs is their capacity to act as microRNA (miRNA) sponges [28]. By binding miRNAs, circRNAs can limit miRNA base-pairing with target mRNAs and thereby reduce miRNA-mediated repression [59]. This competing endogenous RNA (ceRNA) mechanism can indirectly increase expression of miRNA-targeted genes, adding complexity to post-transcriptional regulation [63]. In secondary metabolism, miRNA sequestration may influence the abundance of miRNAs that target transcription factors (e.g., MYB, bHLH) or enzymes (e.g., PAL, CHS), with downstream effects on bioactive compound accumulation [36,38].

Beyond miRNA sequestration, circRNAs have been proposed to regulate transcription [59,60]. Nuclear-localised circRNAs may interact with transcriptional complexes or influence RNA polymerase II activity, affecting transcription of parental or unrelated genes [64]. In medicinal plants, such mechanisms could contribute to coordinated changes in gene expression required for metabolite biosynthesis, particularly during stress responses or developmental transitions. circRNAs have also been suggested to act as scaffolds for RNA-protein complexes, promoting interactions among proteins involved in transcription, RNA splicing or translation [59]. Such scaffolding could support spatial organisation of biosynthetic enzymes and regulatory proteins at metabolon-associated sites and thereby enhance metabolic efficiency.

Evidence is emerging that circRNA expression is dynamically regulated by elicitors and developmental cues in medicinal plants [65]. Elicitors such as methyl jasmonate (MeJA), salicylic acid (SA) and pathogen-associated molecular patterns (PAMPs) are reported to induce extensive transcriptional reprogramming and secondary metabolite production [36,66]. Transcriptomic studies have identified differentially expressed circRNAs in elicitor-treated *Artemisia annua*, *Salvia miltiorrhiza* and *Camellia sinensis*, supporting their potential involvement in signalling networks that regulate defence-associated metabolic reprogramming. circRNA profiles also vary across developmental stages, including root maturation, flowering and leaf senescence, which can coincide with increased accumulation of specific phytochemicals such as alkaloids, flavonoids or terpenoids [67].

Collectively, circRNAs may influence the timing, localisation and coordination of metabolic gene expression by modulating gene regulatory networks through miRNA sequestration and/or protein-interaction scaffolding [59,68]. Such regulation may enable fine control of pathway flux, allowing secondary metabolite output to adjust to environmental and physiological demands [68]. This possibility is relevant for tightly regulated pharmaceutical metabolites, such as vincristine in *Catharanthus roseus* and ginsenosides in *Panax* species [69]. Although evidence linking plant circRNAs to secondary metabolism remains limited, available findings suggest that circRNAs can contribute to phytochemical regulation in medicinal plants. Continued improvements in circRNA detection and functional validation are expected to clarify these roles and inform potential applications in metabolic engineering aimed at improving the yield and consistency of therapeutic natural products.

### 2.16 Metabolic coordination and systemic RNA signaling

RNA-based systemic signalling has emerged as an important regulatory mode that coordinates physiological and metabolic responses across distant plant tissues [70]. Mobile small RNAs, including microRNAs (miRNAs) and small interfering RNAs (siRNAs), function not only as local post-transcriptional regulators but also as long-distance signals that coordinate gene expression beyond their sites of production [26,71]. By moving across cellular boundaries and through vascular tissues, these RNAs can support spatial coordination of secondary metabolism—particularly relevant in medicinal plants, where metabolite biosynthesis and accumulation are often partitioned among organs such as roots, stems and leaves [26,71].

The phloem, a major conduit for assimilates, hormones and signalling molecules, also mediates systemic transport of regulatory RNAs [72]. In phloem sap, small RNAs are stabilised through association with RNA-binding proteins or by packaging into extracellular vesicles, enabling movement between source and sink tissues [72]. Phloem-mediated RNA mobility can therefore contribute to coordinated regulation of metabolite distribution between shoots and roots. For medicinal plants in which roots accumulate high-value metabolites such as *Panax ginseng* and *Salvia miltiorrhiza* long-distance RNA signalling may coordinate expression of key biosynthetic genes in response to environmental or developmental cues perceived in aerial tissues [73]. Nutrient-responsive miRNAs, including miR399 and miR827, have been shown to move systemically and regulate gene expression associated with metabolite biosynthesis, supporting root–shoot coordination of metabolic resource allocation under changing conditions [74]. In addition, small RNAs can be induced rapidly in stressed tissues and redistributed systemically to reprogramme gene expression and secondary metabolism in distal organs during nutrient deprivation, drought or pathogen attack [75]. This systemic response can refine phytochemical biosynthesis across the plant, supporting defence, homeostasis and growth–defence trade-offs. Such coordination is particularly relevant for defence-associated secondary metabolites, including alkaloids, terpenoids and phenylpropanoids, many of which have pharmacological importance.

Systemic RNA networks may also contribute to tissue-specific phytochemical biosynthesis. Enrichment or depletion of specific miRNAs and siRNAs has been observed in particular tissues and correlated with local accumulation of certain metabolites [76]. In *Artemisia annua*, regulatory RNAs transported between leaves and roots have been suggested to influence artemisinin-related gene expression, linking metabolite production to developmental stage and stress status [77]. In *Catharanthus roseus*, coordination of terpenoid indole alkaloid biosynthesis between roots and shoots may likewise involve mobile RNAs that integrate hormonal and stress cues into a coherent metabolic response.

Beyond small RNAs, there is emerging evidence that long noncoding RNAs (lncRNAs) and circular RNAs (circRNAs) may participate in systemic RNA signalling, although their mechanisms remain less well defined [59]. These ncRNAs could contribute to epigenetic memory or longer-term regulation of biosynthetic gene expression, particularly during repeated stress, potentially through mobile RNA-protein or RNA-RNA complexes that transmit regulatory information between tissues [78]. Overall, the systemic mobility of regulatory RNAs provides a framework for coordinating secondary metabolism across spatially separated organs, enabling dynamic responses to developmental programmes and environmental stimuli. In medicinal plants, this signalling may influence both spatial patterning of metabolite biosynthesis and the consistency of therapeutic compound production, with implications for metabolic engineering. Clarifying the routes, specificity and functional consequences of mobile RNA communication will therefore be important for both fundamental plant biology and applied phytopharmaceutical research.

### 2.17 Biophysical and structural considerations

**Fig. 3:**
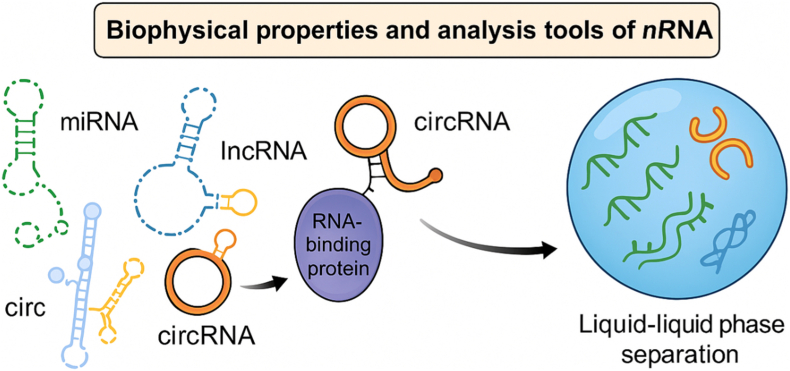
Biophysical properties and analytical approaches for noncoding RNAs.

Understanding ncRNA function in plant secondary metabolism requires not only genomic and transcriptomic perspectives but also consideration of the biophysical and structural properties that underpin RNA-mediated regulation (Fig. 3). RNA stability, subcellular localisation and molecular recognition are shaped by secondary structure formed through intramolecular base pairing. Structural motifs including stem loops, hairpins, bulges and pseudoknots can confer resistance to exonuclease-mediated degradation and influence binding affinity and specificity for complementary RNA targets and protein partners [79]. In the context of secondary metabolism, these features support temporally and spatially resolved regulation of gene expression in response to developmental and environmental cues.

RNA-binding proteins (RBPs) are central determinants of ncRNA specificity in vivo. By interacting with ncRNAs to form ribonucleoprotein (RNP) complexes, RBPs regulate RNA stability, localisation, processing and translation [80]. RBPs recognise sequence and/or structural motifs, enabling selective interactions with miRNAs, long noncoding RNAs (lncRNAs) and circular RNAs (circRNAs) that influence metabolic genes [66]. For example, RBPs have been reported to guide ncRNAs to target transcripts or chromatin sites during regulation of flavonoid and alkaloid pathways, thereby affecting transcript stability or chromatin accessibility [81]. In addition, lncRNAs may act as scaffolds or decoys that sequester RBPs from their canonical targets, indirectly reshaping post-transcriptional regulation of secondary metabolic genes.

Liquid-liquid phase separation (LLPS) is another biophysical phenomenon relevant to RNA-mediated regulation. Through LLPS, RNAs and proteins can assemble into membraneless condensates in the nucleus or cytoplasm, such as stress granules or nuclear bodies, which concentrate specific RNAs and proteins to enable rapid, localised control of gene expression [82]. Phase-separated condensates may support compartmentalisation of biosynthetic regulation in plants, particularly under stress when secondary metabolism is rapidly reprogrammed [83]. In this setting, ncRNA-RBP condensates could coordinate multiple biosynthetic enzymes, transcription factors and associated regulators to enhance the specificity of phytochemical responses [84].

A range of experimental approaches has advanced understanding of RNA structural dynamics and RNA-protein interactions. In vivo RNA structure-mapping methods, including SHAPE (Selective 2′-Hydroxyl Acylation analysed by Primer Extension) and DMS-seq (dimethyl sulfate sequencing), enable interrogation of RNA folding and its modulation by cellular context and environmental cues [85]. These approaches have helped link stress-associated structural changes to regulatory roles in ncRNAs. RNA-protein interaction assays, such as RNA immunoprecipitation (RIP), crosslinking and immunoprecipitation followed by sequencing (CLIP-seq) and enhanced crosslinking (eCLIP), have enabled identification of RNA-RBP networks that post-transcriptionally regulate metabolic pathways [86]. In vivo RNA imaging tools including fluorescence in situ hybridisation (FISH), RNA aptamer-based systems (e.g., Spinach and Mango) and MS2 tagging support real-time observation of RNA localisation and dynamics in plant cells [87]. These methods demonstrate how ncRNA localisation can relate to compartmentalisation of metabolic activities, revealing spatial regulatory layers that were previously difficult to assess.

Taken together, these findings underscore the complexity of RNA-based regulation in plant secondary metabolism. Integrating structure-function relationships, RNA-protein interaction networks and subcellular dynamics shows that ncRNA function extends beyond gene silencing alone [88]. This mechanistic understanding also provides a basis for targeted manipulation of metabolic pathways in medicinal plants using synthetic biology and RNA engineering strategies.

Conceptual schematic showing how distinct classes of noncoding RNAs (ncRNAs) including microRNAs (miRNAs), long noncoding RNAs (lncRNAs) and circular RNAs (circRNAs) adopt diverse secondary structures and interact with RNA-binding proteins to form ribonucleoprotein assemblies. These interactions may promote biomolecular condensate formation through liquid–liquid phase separation, illustrated by the compartmentalisation of heterogeneous RNA species within a droplet-like phase. **Abbreviations:** ncRNA, noncoding RNA; miRNA, microRNA; lncRNA, long noncoding RNA; circRNA, circular RNA.

### 2.18 Applications and biotechnological implications

The emerging recognition of non-coding RNAs (ncRNAs) as key regulators of plant secondary metabolism creates opportunities for biotechnological innovation and metabolic engineering, particularly for the sustainable and precise production of high-value phytochemicals, including alkaloids, flavonoids, terpenoids, and phenolic compounds [89,90]. Owing to their transcriptional and post-transcriptional control of biosynthetic pathways, ncRNAs offer highly specific and tunable regulatory nodes that can be leveraged to increase metabolite accumulation, redirect metabolic flux, or suppress competing pathways in medicinal plants [90]. Targeting ncRNAs in metabolic engineering has been proposed as an alternative approach to address limitations of classical strategies based on gene overexpression or knockout [91].

For example, modulating microRNAs (miRNAs) that target transcription factors such as MYB, bHLH, or WRKY can influence coordinated networks of biosynthetic genes involved in metabolite production [34,36,38,73]. Similarly, long non-coding RNAs (lncRNAs) and circular RNAs (circRNAs) implicated in scaffold formation, chromatin remodelling, and miRNA sponging may be exploited to reprogramme secondary metabolic outputs in a context-dependent manner [92]. Overexpression or silencing of these regulatory RNAs can provide more precise control of phytochemical production with respect to timing, tissue location, and abundance, while limiting unintended effects on plant growth and development.

The advent of CRISPR/Cas-based genome-editing technologies has improved the precision with which ncRNAs can be interrogated and engineered [41]. Compared with conventional transgenic approaches, CRISPR/Cas systems can introduce targeted alterations in ncRNA loci, including promoter elements, processing sites, or binding motifs, thereby enabling functional dissection and fine-tuning of endogenous ncRNA expression [41]. CRISPR/Cas9 and CRISPR/dCas-based transcriptional regulators can be adapted to disrupt or epigenetically silence specific miRNAs or lncRNAs, or to activate their expression without altering the underlying genomic sequence [93]. Experimental models have shown that such targeted modulation can influence flavonoid and terpenoid biosynthesis, providing a practical toolkit for engineering plant metabolic pathways at the level of regulatory RNAs [38].

Beyond genome editing, synthetic RNA tools offer a flexible platform for modular regulation of secondary metabolism. Synthetic lncRNA decoys and artificial miRNAs (amiRNAs) can be designed as regulatory switches or sponges to intercept endogenous signals and redirect metabolite flux towards desired compounds [94]. These constructs can be programmed to respond to developmental cues or external stimuli (e.g., light, temperature, elicitors), adding an environmentally responsive layer of control over biosynthetic gene expression. Spatial and temporal specificity can be further achieved using inducible or tissue-specific promoters to drive synthetic RNA expression, restricting metabolite production to particular organs (e.g., roots, trichomes) or developmental stages and thereby improving yield while reducing metabolic burden [95].

In translational terms, ncRNA biology can be integrated into modern breeding programmes to develop medicinal plant varieties with improved phytochemical traits [96]. Regulatory RNA markers, such as polymorphisms in miRNA genes or target sites, may support marker-assisted selection (MAS) and genomic prediction, enabling selection for optimised metabolic phenotypes. In addition, ncRNA expression profiling during stress responses or developmental transitions may provide biomarkers of plant quality, resilience, and phytochemical content for use in breeding programmes [94]. Overall, ncRNA-based approaches provide a distinct avenue for plant metabolic engineering and may support the development of crops and medicinal plants with enhanced therapeutic metabolite profiles and improved environmental resilience [95]. Integrating RNA biology with synthetic biology, genome editing, and systems-level modelling is likely to be central to realising the utility of ncRNAs in optimising plant metabolism for industrial, pharmaceutical, and nutraceutical applications [96]. This shift towards regulatory RNA-centred, rather than exclusively gene-centred, strategies may reshape approaches to breeding and engineering plants for targeted phytochemical production.

### 2.19 Knowledge gaps and future Direction

Despite substantial advances in understanding ncRNA regulatory functions in plant science, major gaps remain regarding how these molecules regulate secondary metabolite production in medicinal plants [13,14,32,41,58,65,84]. Much of the current evidence derives from model species such as *Arabidopsis thaliana* and *Oryza sativa*; consequently, the functional roles of many lncRNAs and circRNAs in non-model and medicinal plants remain largely uncharacterised [97,98]. Functional validation, particularly for lncRNAs and circRNAs, remains challenging because these transcripts often show pleiotropic, context-dependent and low-abundance expression patterns [99]. Mechanistic understanding of their contributions to metabolite biosynthesis and stress responses is further constrained by experimental limitations, including difficulties in perturbing these RNAs in vivo, frequent off-target effects, and a lack of well-established functional assays.

Prediction and validation of ncRNA-target interactions also remain immature, particularly for specialised metabolic pathways. Although computational tools can predict miRNA-mRNA interactions using sequence complementarity and evolutionary conservation, comparable in silico approaches for identifying lncRNA and circRNA targets are still developing [100]. This difficulty is compounded by the complex regulatory architectures in which these ncRNAs operate, including competing endogenous RNA networks (miRNA sponging), chromatin modification, and RNA-protein interactions [84]. Moreover, many predictions rely on incomplete datasets and there is a need for robust, high-throughput pipelines capable of handling the analytical complexity associated with thousands of ncRNAs [61,62].

Addressing these gaps will require integrated multi-omics approaches, including transcriptomics, metabolomics, epigenomics, and small RNA profiling, to resolve ncRNA-dependent regulation of secondary metabolism [101]. In addition to identifying co-expression modules that link ncRNAs with metabolite accumulation, multi-omics integration can help delineate regulatory circuits underlying environmental and developmental control of phytochemical biosynthesis [102]. For instance, comparing metabolite profiles across tissues, stress conditions, or developmental stages may reveal candidate regulatory axes associated with ncRNA expression [65,84]. Incorporating epigenomic information (DNA methylation and histone modifications) may further clarify whether subsets of lncRNAs mediate chromatin state changes at biosynthetic gene loci, improving understanding of how epigenetic landscapes contribute to metabolic plasticity [24,[103], [104], [105], [106], [107], [108], [109]].

Progress will also depend on developing species-specific ncRNA resources for medicinal plants. While databases such as miRBase and NONCODE contain valuable information for model organisms, coverage of medicinal plants many of which possess specialised and diverse metabolic repertoires remains limited [[110], [111], [112]]. Dedicated databases capturing sequence features, expression profiles, predicted and validated targets, structural motifs, and associated metabolites for species such as *Artemisia annua*, *Salvia miltiorrhiza*, and *Catharanthus roseus* would facilitate discovery and hypothesis testing. Including environmental and stress-response metadata would further strengthen functional annotation across physiological contexts.

Future work should prioritise experimental validation platforms tailored to medicinal plant systems, including CRISPR/Cas-mediated disruption, RNA interference, and transient overexpression approaches. In parallel, computational advances, including machine learning-based predictive models, should be used to integrate multi-omics data and infer ncRNA function. Comparative analyses across species may help distinguish conserved regulators from lineage-specific ncRNAs. As single-cell RNA-seq and spatial transcriptomics mature, these approaches should enable resolution of cell-type-specific ncRNA expression patterns and their contributions to tissue-specific metabolic regulation.

In summary, while understanding of ncRNA regulatory roles in plant secondary metabolism is advancing, substantial knowledge gaps must be addressed to realise their biotechnological potential. Coordinated efforts combining functional genomics, multi-omics integration, species-specific resources, and advanced bioinformatics will be essential to map ncRNA regulatory landscapes in medicinal plants and to translate this knowledge into metabolic engineering and plant-based drug production.

## 3 Recommendations and future perspectives

### 3.1 Move from association to causality in medicinal plants

Prioritise CRISPR/Cas-based perturbation of ncRNA loci and validated target sites, coupled with quantitative metabolomics, to establish causal relationships rather than correlations. This shift is particularly important for lncRNAs and circRNAs, for which functional evidence remains limited.

### 3.2 Standardise evidence reporting and transparency

Adopt minimal reporting standards for ncRNA-metabolite studies, including the validation assay type(s), number of biological replicates, metabolite quantification platform and normalisation strategy, and off-target assessment. Deposit analysis pipelines, code, and where feasible processed datasets to support reproducibility and reuse.

### 3.3 Develop medicinal-plant ncRNA resources

Establish species-resolved ncRNA atlases that integrate expression profiles, structural features, experimentally validated targets, metabolite outputs, and stress conditions. Such resources are required to improve cross-study comparability and to enable reproducible engineering.

### 3.4 Integrate multi-omics with spatial resolution

Combine small-RNA profiling with epigenomics and metabolomics, and incorporate single-cell and/or spatial transcriptomics where feasible, to resolve tissue- and cell-type-specific regulation of secondary metabolite pathways.

### 3.5 Engineer regulatory RNA circuits for precise pathway control

Apply synthetic amiRNAs, lncRNA decoys, and inducible RNA modules to achieve tissue- and time-specific tuning of pathway flux, potentially with fewer developmental trade-offs than constitutive enzyme overexpression.

### 3.6 Strengthen sustainability and translational linkage

ncRNA-guided metabolic engineering may stabilise phytochemical supply chains and reduce pressure from overharvesting. In parallel, complementary therapeutic strategies targeting metabolic pathways such as incretin/GLP-1 axis pharmacology (e.g., DPP-4 inhibition) and proteasome-targeting approaches highlight broader translational opportunities, while plant-derived compounds (e.g., hinokitiol) remain of interest in immunomodulatory and anticancer contexts.

## 4 Conclusion

Non-coding RNAs (ncRNAs) form a multilayered regulatory architecture that controls the timing, tissue specificity, and magnitude of specialised metabolite biosynthesis in medicinal plants. MicroRNAs (miRNAs) typically act through transcription factor hierarchies and, in some cases, by directly targeting biosynthetic enzymes. Small interfering RNAs (siRNAs) contribute via RNA-directed DNA methylation (RdDM) and transcriptional silencing. Long non-coding RNAs (lncRNAs) and circular RNAs (circRNAs) extend regulation to chromatin and network levels through scaffolding, decoy activity, and competing endogenous RNA (ceRNA) interactions. However, the strength of evidence varies across ncRNA classes and plant systems, and causal validation remains limited for many medicinal species. Closing these gaps will require rigorous and reproducible study appraisal, standardised reporting, and integrated multi-omics designs to support ncRNA-informed metabolic engineering and breeding strategies for the sustainable production of therapeutic phytochemicals.

## Consent to participate

Not applicable.

## Consent to publish declaration

Not applicable.

## Ethics approval

Not applicable.

## Clinical trial date of registration

Not applicable.

## Clinical trial registration number

Not applicable.

## Clinical trial registry

Not applicable.

## Generative AI disclosure statement

During the preparation of this work, the author(s) used **QUILBOT** to **improve scientific language, clarity, and readability.** After using this tool, the author(s) reviewed and edited the content as needed and take full responsibility for the content of the published article.

## Funding

None.

## CRediT authorship contribution statement

**Okechukwu Paul-Chima Ugwu:** Conceptualization, Investigation, Methodology, Supervision, Validation, Visualization, Writing – original draft, Writing – review & editing. **Melvin Nnaaemeka Ugwu:** Conceptualization, Supervision, Validation, Visualization, Writing – original draft, Writing – review & editing. **Hope Onohuean:** Conceptualization, Data curation, Supervision, Validation, Visualization, Writing – original draft, Writing – review & editing. **Hilal Ahmad Rather:** Supervision, Validation, Visualization, Writing – original draft, Writing – review & editing. **Ibe Michael Usman:** Supervision, Validation, Visualization, Writing – original draft, Writing – review & editing.

## Declaration of competing interest

The authors declare that they have no known competing financial interests or personal relationships that could have appeared to influence the work reported in this paper.

## Data Availability

Data will be made available on request.
