# Supplementary material for: Noncoding RNAs and the phytochemical economy: Molecular regulators of secondary metabolism in medicinal plants
Source: Biochem Biophys Rep. 2026 Feb 9;45:102486. doi: 10.1016/j.bbrep.2026.102486 (PMC12907886; doi:10.1016/j.bbrep.2026.102486)
Supplement: Multimedia component 1 [file mmc1.docx]

**Table S1: Information sources, core search strings and limits used in the systematised narrative review.**

| **Database** | **Core search strategy (concept blocks combined with AND)** | **Limits / notes** |
| --- | --- | --- |
| PubMed/MEDLINE | (“non-coding RNA” OR ncRNA OR microRNA OR miRNA OR “small interfering RNA” OR siRNA OR “long non-coding RNA” OR lncRNA OR circRNA OR “circular RNA”) AND (“secondary metabolism” OR “specialised metabolism” OR phytochemical* OR alkaloid* OR flavonoid* OR terpenoid* OR phenylpropanoid*) AND (“medicinal plant*” OR herb* OR “plant biotechnology” OR “metabolic engineering”) | English language; plant-focused studies |
| Scopus | TITLE-ABS-KEY((non-coding RNA OR ncRNA OR miRNA OR siRNA OR lncRNA OR circRNA) AND (“secondary metabolism” OR “specialised metabolism” OR phytochemical* OR alkaloid* OR flavonoid* OR terpenoid* OR phenylpropanoid*) AND (“medicinal plant*” OR herb* OR “plant biotechnology” OR “metabolic engineering”)) | Articles and reviews |
| Web of Science Core Collection | TS=(non-coding RNA OR ncRNA OR microRNA OR miRNA OR siRNA OR lncRNA OR circRNA) AND TS=(secondary OR specialised metabolism OR phytochemical* OR alkaloid* OR flavonoid* OR terpenoid* OR phenylpropanoid*) AND TS=(medicinal plant* OR herb* OR plant biotechnology OR metabolic engineering) | Research articles and reviews |
| CAB Abstracts (CABI) | (non-coding RNA OR ncRNA OR miRNA OR siRNA OR lncRNA OR circRNA) AND (secondary metabolism OR specialised metabolism OR phytochemical*) AND (medicinal plant* OR herb*) | No geographic restriction |
| AGRICOLA | (non-coding RNA OR microRNA OR miRNA OR siRNA OR lncRNA OR circRNA) AND (secondary metabolism OR phytochemical*) AND (medicinal plant*) | English language |

**Supplementary searching:** Google Scholar (first 200 results per query string) and manual reference list screening of high-relevance reviews and seminal mechanistic studies.

**Table S2: Summary of literature identification, screening, qualitative appraisal and thematic synthesis in this SANRA-guided narrative review**

| **Review stage** | **Description** | **Records / studies (n)** |
| --- | --- | --- |
| **Literature identification** | Records retrieved from bibliographic databases | 1,284 |
|  | Records identified from supplementary sources | 216 |
|  | **Total records identified** | **1,500** |
| **De-duplication** | Duplicate records removed | 412 |
|  | **Records after de-duplication** | **1,088** |
| **Title and abstract screening** | Records screened | 1,088 |
|  | Records excluded | 792 |
|  | **Records retained for full-text assessment** | **296** |
| **Full-text assessment** | Full-text articles assessed | 296 |
|  | Full-text articles excluded | 198 |
|  | – No mechanistic link to specialised metabolism | 86 |
|  | – ncRNA reported without functional or pathway relevance | 54 |
|  | – Expression profiling only | 38 |
|  | – Review or commentary without primary data | 20 |
| **Qualitative synthesis** | Studies included in narrative synthesis | **98** |
| **Quality appraisal (SANRA-aligned)** | High quality (direct ncRNA–target validation with metabolite evidence) | 41 |
|  | Moderate quality (partial molecular validation and/or metabolite measurement) | 37 |
|  | Low quality (correlational or predictive evidence only) | 20 |
| **Thematic contribution** | Studies contributing to primary mechanistic themes | 78 |
|  | Studies retained for contextual or hypothesis-generating insight | 20 |

Study quality tiers informed interpretive weighting in the narrative synthesis but were not used as exclusion criteria, consistent with SANRA guidance for narrative reviews. Counts are provided to ensure transparency without framing the review as a formal systematic review or meta-analysis.
